# ISO 9001 certification for hospitals in Bulgaria: does it help service?

**DOI:** 10.1080/13102818.2014.915491

**Published:** 2014-07-04

**Authors:** Assena Stoimenova, Ani Stoilova, Guenka Petrova

**Affiliations:** ^a^ Department of Social Pharmacy and Pharmacoeconomics, Faculty of Pharmacy, Medical University, Sofia, Bulgaria; ^b^ Certification Division, RINA Bulgaria Ltd., Sofia, Bulgaria

**Keywords:** ISO 9001, quality management, hospitals, implementation, effectiveness

## Abstract

The aim of our study is to review the published literature on establishment and implementation of ISO 9001 QMS in European hospitals, to study the availability of International Organization for Standardization (ISO) quality management systems (QMS) in Bulgarian hospitals and to outline the main advantages of ISO implementation in the hospitals in Bulgaria. The information on availability of ISO QMS in the hospitals in Bulgaria was gathered via Bulgarian certification register, the registries of various quality associations, websites of hospitals and certification companies presented in Bulgaria. A total number of 312 hospitals in Bulgaria were screened for the availability of QMS certified against the ISO 9001 requirements. The experience of European hospitals that implemented QMS is positive and the used approaches to improve the processes and the demonstrated effects from ISO implementation are analysed by the researchers. Unlike other European Union member states, the establishment of quality management systems in Bulgaria is not compulsory. However, our study revealed that 14.42% of the hospitals in Bulgaria have implemented and have certified quality systems against the requirements of ISO 9001. Our study confirmed that a quality management system using the ISO 9001 standard is useful for the hospitals as it can help to increase the operational efficiencies, to reduce errors, improve patient safety and produce a more preventive approach instead of a reactive environment.

## Introduction

Health care organizations are expected to deliver a high-level quality of care. Moreover, the society demands efficient usage of public funding, transparency and accountability.[1–4] Since the 1990s, there is a general trend for stakeholders to put more pressure on hospitals for equity of access to health, accountability and transparency. That is one of the reasons why the governments of some countries in the European Union (EU) have stimulated the implementation of various quality management systems (QMS).[2,4–7] Most of them are based on International Organization for Standardization (ISO) certification. Initially, ISO standards were focused on technical specifications geared for manufacturing and scientific industries but later, with the creation of the ISO 9001 QMS, their scope expanded and addressed a broad range of business processes, applicable to almost any kind of organization in any industry, including hospitals.[2,8–13] Some ISO standards align well with the operations of hospitals as they promote the use of practices that protect safety and privacy of patients [6,14–20] and the continuous process of improvement, one of the fundamental principles of ISO 9001, which subsequently creates ways to improve the comfort of patients.[4,21,22]

Some countries (e.g. Greece, Portugal and the United Kingdom) have no legal requirement for hospitals to meet specific organizational standards, whereas in other countries (e.g. Germany, France and Austria) governments have legislated some form of internal and/or external assessment of hospital services based on ISO certification.[18,19,23,24]

The aim of our study is to review the published literature on establishment and implementation of ISO 9001 QMS in European hospitals, to study the availability of ISO QMS in Bulgarian hospitals and to outline the main advantages of ISO implementation in the hospitals in Bulgaria.

The main study questions were the following:

1. What are the available published evidences for ISO implementation in the European hospitals?
2. How many hospitals in Bulgaria are ISO certified and what are the obstacles and benefits of such certification?

## Materials and methods

Published scientific literature about the availability of ISO certification in European hospitals were obtained after Internet search of Scopus, ISI Web of Knowledge and ScienceDirect databases with the following key words: ‘ISO certification’, ‘quality management systems’, ‘hospitals’ and ‘European Union’. On total 53 articles were found and 33 were discussed in this study. The information from the articles was used to formulate key questions addressing the Bulgarian hospital managers’ opinion on establishment, implementation and certification of quality management systems in hospitals according to ISO.[1–5,7–36]

The information on availability of ISO QMS in the hospitals in Bulgaria was gathered via Bulgarian certification register,[37] the registries of various quality associations,[38] websites of hospitals and certification companies presented in Bulgaria. A total number of 312 hospitals in Bulgaria were screened for the availability of QMS certified against the ISO 9001 requirements.

Opinion of hospital managers regarding the obstacles met during the preparation phase and the outcomes from ISO 9001 certification were gathered by the means of interviews. We have interviewed by e-mail the executive directors or management representatives of the 45 ISO 9001 certified hospitals and received 13 questionnaires that were completely filled in (response rate of 28.89%). The statements included into the interview were categorized according to a five-point Likert scale (1: strongly disagree, 2: disagree, 3: neither agree nor disagree, 4: agree and 5: strongly agree).

## Results and discussion

### Review of published literature on development of QMS in European hospitals

ISO 9001 certification is utilized in a variety of ways as a vehicle for health care organizations to identify systemic breakdowns and close gaps, streamline workflow and maximize resource utilization, focus on patient and provider needs and expectations, facilitate compliance to health care accreditation standards and regulatory requirements, etc. [3,4,22]

The Academic Hospital in Utrecht was the first hospital according to the literature that succeeded to implement an ISO 9000 QMS in its radiology department (January 1996).[35] This ISO certification was inspired by the increasing demand for radiological investigations combined with decreasing budgets, the need for an integral and systematic approach and last but not least the Dutch Government had enacted a law which obliged health care organizations in the Netherlands to have a quality control system.[34] According to this legislation all care institutions should provide clarity on the quality management activities by publishing an annual quality report, sent to the Ministry of Health, the Health Inspectorate and the regional patient organizations. Hospitals in the Netherlands are not financially compensated for the implementation of QMS by health insurance funds or the Ministry of Health,[29] unlike the Hungarian hospitals which received financial support for implementation of ISO 9002.[22]

In Hungary, the ‘Act CLIV of 1997 on Health’ regulates the professional requirements for health care services and makes the operation of a QMS obligatory for every hospital.[25] The aims of QMS according to Hungarian legislation are: to continuously improve quality; to explore and plan the process of service; to prevent possible mistakes; to reveal insufficiencies of delivery in time and to take and control necessary measures; to explore the causes of insufficiencies; to decrease damages and expenses incurred by these; to meet professional and quality requirements; and to develop the institutions’ own requirements.

In Finland four national recommendations on establishment of QMS in the hospitals have been issued during 1994–1999. The recommendations are organized according to eight topics: customer participation in quality management; leadership for the steering of quality; personnel as a prerequisite for high quality; preventive nature of quality management activities; management of processes as a basis for QM; information as a basis for the continuous enhancement of quality; systematization of QM and detailed recommendations; and quality criteria to support quality management.[22] Hospitals in Finland use several QM models and criteria for the development of their QMS. The Finnish Quality Award based on the European Foundation of Quality Management (EFQM) model is used in about 50% of the hospitals as are ISO 9000 standards. The Finnish health care accreditation model, based on the King's Fund model (UK) is used in about 20% of central, regional and private hospitals.[24] One of the ISO-certified hospitals in Finland was Kuopio University Hospital which gained formal ISO 9002 certification in 1999.[31]

Duckers et al. [14] studied the developmental stages of the quality management system in Dutch hospitals between 1995 and 2007. Since 1995, hospital quality management systems have reached higher levels of development. Participants in multi-layered programs have developed their QMS more rapidly compared to non-participants. Study results suggest that the combination of policy measures at macro level was accompanied by an increase in hospital size and the further development of quality management systems.

Ennis and Harrington reported the findings from a quantitative research study of quality management in the Irish health care sector.[28] The study findings suggested that quality management is required for hospitals to become more cost-effective. The authors recommended a shift from the traditional management structures to a more participative approach.

Another publication discussed major difficulties in implementing ISO 9001 in a German ophthalmology hospital in 1999, namely translating the industrial norms to the context of an eye hospital and overcoming scepticism towards quality-assurance measures that lie beyond the ophthalmological quality control.[5]

Beholz et al. [26] describes the introduction of a QMS according to ISO 9001 in a university cardiovascular surgery department, its 18 months development and subsequent certification. The researchers evaluated and optimized all necessary resources, through measurement of customer satisfaction using patients’ and referring physicians’ surveys.

Shaw et al. [3] analysed the compliance with measures of quality in 89 hospitals in six countries, as assessed by external auditors using a standardized tool, as part of the European Commission (EC)-funded Methods of Assessing Response to Quality Improvement Strategies project. Of the 89 hospitals selected for external audit, 34 were accredited (without ISO certification), 10 were certificated under ISO 9001 (without accreditation) and 27 had neither accreditation nor certification. Overall percentage scores for 229 criteria of quality and safety were 66.9, 60.0 and 51.2, respectively. Analysis confirmed statistically significant differences comparing mean scores by the type of external assessment (accreditation, certification or neither); however, it did not substantially differentiate between accreditation and certification only. Some of these associations with external assessments were confounded by the country in which the sample hospitals were located. The study confirmed that quality and safety structures and procedures are more evident in hospitals with either the type of external assessment and it suggested that some differences exist between accredited versus certified hospitals. Interpretation of these results, however, is limited by the sample size and confounded by variations in the application of accreditation and certification within and between countries.

The research of van den Heuvel et al. [36] described how The Red Cross Hospital in Beverwijk, the Netherlands, implemented an ISO 9000 QMS and outlined the main advantages from using ISO (re-established focus on patients, introduction of performance measurements, improved quality of care, etc.). In addition, positive effects on patient safety were demonstrated through comparison with 10 other hospitals.

In 2006, Wagner et al. [22] compared the implementation of QMS in the hospitals in the Netherlands, Hungary and Finland, based on an empirical data gathered by a self-administered questionnaire. Data were received from 101 hospitals in the Netherlands, 116 hospitals in Hungary and 59 hospitals in Finland. A mean of 22 QM activities per hospital was found in the Netherlands and Finland versus 20 QM activities in the Hungarian hospitals. Only a small number of hospitals have already implemented a QMS (4% in the Netherlands, none in Hungary and 3% in Finland). More hospitals in the Netherlands concentrated their efforts on quality documents, whereas Finnish hospitals were focused on training in QM and guidelines. Cyclic quality improvement activities have been developed in the three countries, but in most hospitals the results were not used for improvements. All three countries paid hardly any attention to patient participation. Wagner et al. did not confirm the hypothesis that governmental legislation or financial reimbursement can stimulate the implementation of QM activities, more than voluntary recommendations. However, the results of their study showed that specific obligations can stimulate the implementation of QM activities more than general, framework legislation.

Buciuniene et al. [27] reported that QMS were found operating in 39.7% of support treatment and nursing hospitals in Lithuania and under implementation in 46.6% of hospitals (13.7% at the time of the study did not have any QMS). The most critical issues related to the QMS implementation include procedure development, lack of financial resources and information, and development of work guidelines while improved responsibility and power sharing, better service quality and higher patient satisfaction were perceived by the respondents as the key QMS benefits.

In 2009, Lombarts et al. [30] described how EU hospitals have applied seven quality improvement strategies previously defined by the Multi-Center Medication Reconciliation Quality Improvement Study (MARQuIS) study: organizational quality management programs; systems for obtaining patients’ views; patient safety systems; audit and internal assessment of clinical standards; clinical and practice guidelines; performance indicators; and external assessment. A Web-based questionnaire was used to survey acute care hospitals in eight EU countries. Data collection took place from April to August 2006. A total of 389 hospitals participated in the survey. The study confirmed that all seven quality improvement strategies were widely used in the European countries. Activities related to external assessment were the most broadly applied across Europe, and activities related to patient involvement were the least widely implemented. No country implemented all quality strategies at all hospitals. There were no differences between participating hospitals in Western and Eastern European countries in regard to the application of quality improvement strategies.

### ISO certification of hospitals in Bulgaria

The number of Bulgarian hospitals has witnessed a substantial growth over the period 2001–2010. Currently there are 312 registered hospitals in Bulgaria (Table 1).[39,40] There are various kinds of hospitals ranging from general (multi-profile) hospitals to specialized hospitals, governmental hospitals, private hospitals, etc. (Table 1). Most of the major specialized hospitals are located in the capital Sofia. A total of 164 (52.56%) of the Bulgarian hospitals are multi-profile hospitals (general hospitals). The number of specialized hospitals is also quite substantial – 148, and it has remained stable through the observed period.

**Table 1.** Hospitals in Bulgaria (adapted from National Statistical Institute).

Hospitals	2001	2002	2003	2004	2005	2006	2007	2008	2009	2010
Total number of hospitals of which:	244	251	249	257	262	270	292	305	306	312
Multi-profile hospitals	140	140	135	140	141	139	152	157	159	164
Specialized hospitals	102	109	112	117	121	131	140	148	147	148
Dermato-venerological centres	12	12	12	11	10	10	10	10	10	10
Complex oncological centres	12	12	12	11	12	12	12	12	12	9
Mental health centres	12	12	12	12	12	12	12	12	12	12
Pulmonary dispensaries	13	13	13	12	12	12	12	12	12	–
Other establishments for hospital aid	–	–	–	–	–	–	–	–	–	3

Out of 312 hospitals, only 45 (14.42%) are certified according to ISO 9001 requirements (Table 2). All hospitals obtained a certificate for the entire organization.

**Table 2.** ISO 9001 certified hospitals in Bulgaria (first certification): main figures.

	2004	2005	2006	2007	2008	2009	2010	2011
Total number of ISO-certified hospital: 45 (14.42%)	1	3	6	3	9	10	9	4
Multi-profile hospitals	–	1	5	2	7	6	8	4
Specialized hospitals	–	2	1	1	1	1	1	–
Dermato-venerological centres	–	–	–	–	–	–	–	–
Complex oncological centres	–	–	–	–	–	1	–	–
Mental health centres	–	–	–	–	–	1	–	–
Pulmonary dispensaries	–	–	–	–	1	1	–	–
Other establishments for hospital aid	–	–	–	–	–	–	–	–

The first hospital certified against the ISO requirements was a specialized cardiology hospital for active treatment, ‘St. Ekaterina’ in Sofia, which received the certification in June 2004. Later, in early 2008 the hospital received the European Quality Award from the European Business Association. Today the hospital holds a leading position among cardiology hospitals and surgery centres in Bulgaria and Eastern Europe.[41] According to the executive director of ‘St. Ekaterina’ hospital, the most difficult part of their preparation for ISO certification seemed to be teaching people to get used with the various documents and records. The hospital key personnel noticed improvement of the hospital management after establishment and implementation of ISO 9001 management system.[32]

In 2005, one general and two specialized hospitals (a mental health centre and a complex oncological centre) were ISO 9001 certified. In 2006, the number of hospitals that received certification doubled and later on, for a three-year period (2008–2010), 29 hospitals (64.44%) were certified. Of these, 21 were general hospitals and 3 were specialized hospitals. Some of the hospitals’ executives shared the opinion that the needed financial resources for the phase of preparation, the certification and the surveillance audit fees is one of the main reasons but surely not the only one for the low number of certified hospitals.[32,42] Some of the hospitals established and implemented integrated quality systems, incorporating the requirements of several ISO standards – i.e. ISO 9001, ISO 14001 and ISO 18001. An example of such a hospital is the multi-profile hospital ‘St. Panteleimon’ in Plovdiv. The successful certification against the three standards was announced in November 2008.[43]

A number of advantages were pointed out during the interviews with the executive directors or management representatives of the hospitals that implemented ISO 9001 (Table 3). All 13 interviewees shared an opinion that with the ISO 9001 certification patient focus has been re-established. Moreover, the identification of processes and introduction of performance indicators contributed to the increase of effectiveness and also ISO principles helped to support the hospitals’ mission. All of the responders (100%) considered that during the preparation for ISO certification, a documentation system was approved and it did not lead to bureaucracy. At the same time the responders shared that during the preparation they experienced some difficulties (Table 4) such as the specificity of ISO 9001 terminology, increased workload, unavailability of trained internal auditors, selection of a consultant and the time needed for preparation.

**Table 3.** Some benefits of ISO certification.

Statement	5	4	3	2	1
ISO 9001 is a useful tool to implement a quality management system in a hospital	*N* = 10	*N* = 3	–	–	–
Patient focus was re-established	*N* = 13	–	–	–	–
All important processes were identified and they were subject to continuous improvement	*N* = 12	*N* = 1	–	–	–
ISO principles helped to support hospital's mission	*N* = 13	–	–	–	–
Patient safety has improved	*N* = 9	*N* = 2	*N* = 2	–	–
Discovery of causes of poor performance was made easier	*N* = 9	*N* = 3	–	–	–
Customer complaints were reduced	*N* = 5	*N* = 2	*N* = 5	–	–
Documentation system was improved	*N* = 13	–	–	–	–

**Table 4.** Some difficulties concerning the preparation for ISO certification.

Statement	5	4	3	2	1
ISO terminology was not easy to get used with	*N* = 10	*N* = 1	*N* = 1	–	–
Selection of consultant wasn't an easy task	*N* = 4	*N* = 4	*N* = 1	*N* = 4	–
Consultants were more experienced in ISO certification for other types of business (non-health care)	*N* = 9	*N* = 1	*N* = 1	*N* = 1	*N* = 1
No availability of trained internal auditors	*N* = 10	*N* = 1	*N* = 2	–	–
Document workload increased	*N* = 9	*N* = 3	*N* = 1	–	–
Preparation was time consuming process	*N* = 4	*N* = 6	*N* = 1	*N* = 1	*N* = 1

**Figure 1.**
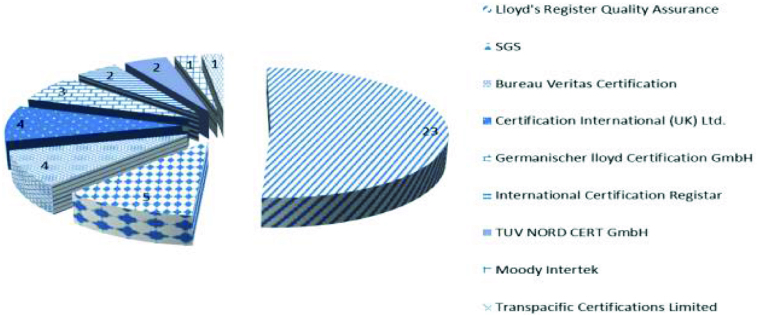
Certification bodies involved in hospital certification.

The certification bodies involved in certification of hospitals’ QMS in Bulgaria are shown in Figure 1. The ultimate leader is Moody Intertek (23 hospitals), followed by SGS (5), Bureau Veritas Certification (4) and Certification International (UK) Ltd. (4).

The review of the literature concerning the establishment and certification of QMS according to ISO 9001 showed that the experience of European hospitals that implemented QMS is positive and the researchers analyse the approaches that are used to improve the processes and the demonstrated effects from ISO implementation.

Unlike other EU member states, in Bulgaria the establishment of quality management systems is not compulsory.[33] However, our study showed that only 14.42% of the hospitals in Bulgaria have implemented and have certified quality systems against the requirements of ISO 9001 (despite the fact that they require their subcontractors to prove their ISO 9001 certification). Some of the hospitals established integrated quality systems matching the ISO 9001 requirements with the ISO 14001 (environmental management standard) and ISO 18001 (occupational health and safety standard). The implementation of quality management in health care according to ISO standards is much slower than in other areas such as trade and production. A possible explanation could be the traditionalism in medical identity, specificity of health services as well as a little knowledge about ISO and its advantages that exists in the medical society.

The identified advantages of ISO certification revealed high satisfaction from the implemented quality management systems which led to re-establishment of patient focus, improvement of patient safety and documentation, increased efficiency, etc. However, some difficulties in establishments and certification of quality management systems were shared by the interviewed executive directors and management representatives such as increased workload, specific terminology, lack of trained auditors, etc. Our study confirmed that a quality management system using the ISO 9001 standard is useful for the hospitals as it can help to increase operational efficiencies, reduce errors, improve patient safety and produce a more preventive approach instead of a reactive environment.

## Conclusions

In today's competitive environment, the quality management processes are proved as successful tools for improving the quality of care and controlling costs in the hospitals. However, these management systems are still not sufficiently popular amongst the majority of the Bulgarian health specialists. Logically, as the hospitals require ISO 9001 certification from their subcontractors they should also take some actions to establish and implement quality management systems according to ISO 9001. The recognition of the need to improve the quality of service that is provided to their patients, more hospitals in Bulgaria seek information from the certifying organizations in order to implement the requirements of ISO 9001:2008 to their activities. Based on the results of our study we do recommend implementation of ISO QMS in the hospitals in Bulgaria as a proven mechanism that improves the quality of patient care and safety.
